# Phytotoxic Effects of Polyethylene Microplastics on the Growth of Food Crops Soybean (*Glycine max*) and Mung Bean (*Vigna radiata*)

**DOI:** 10.3390/ijerph182010629

**Published:** 2021-10-11

**Authors:** Lin Wang, Yi Liu, Mandeep Kaur, Zhisheng Yao, Taizheng Chen, Ming Xu

**Affiliations:** 1 Department of Environmental Science, College of Geography and Environmental Science, Henan University, Kaifeng 475004, China; wanglin@henu.edu.cn (L.W.); mk9041985@gmail.com (M.K.); 2 Department of Environmental Science, Miami College, Henan University, Kaifeng 475002, China; liuyiliuzimo@163.com; 3 Henan Key Laboratory of Earth System Observation and Modeling, Henan University, Kaifeng 475004, China; 4 State Key Laboratory of Atmospheric Boundary Layer Physics and Atmospheric Chemistry, Institute of Atmospheric Physics, Chinese Academy of Sciences, Beijing 100029, China; zhishengyao@mail.iap.ac.cn

**Keywords:** micro-plastics particles, phyto-toxicity, agricultural crop, root length, seed vigor, seed germination

## Abstract

Accumulation of micro-plastics (MPs) in the environment has resulted in various ecological and health concerns. Nowadays, however, studies are mainly focused on toxicity of MPs on aquatic organisms, but only a few studies assess the toxic effects of micro-plastics on terrestrial plants, especially edible agricultural crops. The present study was aimed to investigate the adverse effects of polyethylene (PE) microplastics on the germination of two common food crops of China, i.e., soybean (*Glycine max*) and mung bean (*Vigna radiata*). Both the crops were treated with polyethylene microplastics (PE-MPs) of two sizes (6.5 μm and 13 μm) with six different concentrations (0, 10, 50, 100, 200, and 500 mg/L). Parameters studied were (i) seed vigor (e.g., germination energy, germination index, vigor index, mean germination speed, germination rate); (ii) morphology (e.g., root length, shoot length) and (iii) dry weight. It was found that the phyto-toxicity of PE-MPs to soybean (*Glycine max*) was greater than that of mung bean (*Vigna radiata*). On the 3rd day, the dry weight of soybean was inhibited at different concentrations as compared to the control and the inhibition showed decline with the increase in the concentration of PE-MPs. After the 7th day, the root length of soybean was inhibited by PE-MPs of 13 μm size, and the inhibition degree was positively correlated with the concentration, whereas the root length of mung bean was increased, and the promotion degree was positively correlated with the concentration. Present study indicated the necessity to explore the hazardous effects of different sizes of PE-MPs on the growth and germination process of agricultural crops. Additionally, our results can provide theoretical basis and data support for further investigation on the toxicity of PE-MPs to soybean and mung bean.

## 1. Introduction

The occurrence of micro-plastics (MPs) in all three forms of ecosystems viz., air, water and soil have gradually drawn more attention in scientific community in recent years. MPs are usually defined as plastic debris particles with a diameter ≤5 mm [1,2,3]. MP is generated when plastics present in the environment are converted into small fragments with the action of heat, weathering, UV radiation, mechanic abrasion and bio-degradation [2,4,5,6,7]. Due to the action of heat/UV rays, plastic lead to the production of free radicals via photo oxidative degradation. It releases chemicals like dioxins, phthalates, poly vinyl chloride, bis-phenols, heavy metals like antimony lead, cadmium, etc. [8,9]. MPs then migrate in the environment via atmospheric circulation and ocean currents, resulting in their abundant accumulation in the major sinks such as estuaries, rivers, lakes, oceans, organisms, etc. [6,10,11,12]. Accumulation of MPs in water bodies, sediments, soil and organisms was reported widely in many earlier studies [10,11,13,14]. Due to high hydro-phobicity and large surface area of MPs, they have become carrier for pathogens and many other pollutants [15,16]. Additionally, they have been ingested by many organisms through food chain, which results in negative effects on ecosystem and human health [1,17,18]. Therefore, the pollution caused by MPs and their eco-toxicological effects need more researches in future.

Among many chemicals released from MPs, bis-phenol A (BPA) and phthalates are well known compounds, if inhaled or ingested can led to endocrine disruption. BPA is a common plasticizer used in food packaging and polycarbonate plastics manufacturing industry [19,20]. In an open environment, bio-degradation of BPA compounds by extracellular and intracellular fungal enzymes (peroxidases, laccase, cytochrome P450 mono-oxygenases) is well documented [21,22]. BPA being persistent and unstable in nature can facilitate its leaching, and thereby high absorption in the aquatic environments [23]. The presence of BPA (1–729.9 ng/g) on MPs was first time studied in samples collected from the open, remote oceans and urban beaches from America and Europe [24]. Another study by Rehse et al. [25] showed the effects of BPA present in non-suspended (aggregated at the water surface) polyamide MP particles on freshwater zooplankton (*Daphnia magna*) and its absorption in plastic fragments from remote coasts and open ocean shores. MPs are generally classified into two types based on their origin [26]. MPs generated through grinding are known as primary MPs for, e.g., drug carriers and abrasives used in personal care products such as shampoos, tooth pastes and cleansers [13,27]. On the other hand, secondary MPs are formed under the external force of physical, chemical and biological degradation of large and visible plastic items such as commodity packages, discarded clothing, digital products, footwear, plastic bottles and fibers in laundry effluents [12,27,28,29]. In addition, based on polymer type, micro-plastics can be polyethylene (PE), polypropylene (PP), polyvinyl chloride (PVC), polystyrene (PS), polyamide (PA), poly (ethylene terephthalate) (PET), poly-lactic acid (PLA), etc. [30,31].

In the literature, many studies have been conducted to evaluate the impact of MPs to terrestrial agricultural plants [31,32,33,34,35,36,37,38]. It was reported that the absorption of MPs to wheat (*Triticum aestivum*) was dimensional dependent on the size of MPs [31]. In detail, the experiment confirmed that MPs less than 36 nm could be transferred easily from root to leaf; MPs between sizes 40 nm and 140 nm were retained in root; and MP particles larger than 140 nm could not be absorbed [31]. Another study confirmed that the effects of PE, PP, and PET MPs on spring onion (*Allium fisculosum*) were not significant, while PS increased the root biomass and PA decreased the stem biomass [32]. Furthermore, it was observed that the germination rate of cress (*Lepidium sativum*) was inhibited after 8 h exposure to three sizes of MPs (50, 500 and 4800 nm). The experimental results indicated that cress seeds could bio-accumulate MPs during seed germination and forming a physical barrier that reversely hindered the root growth [33]. Zhang et al. in their study proved the in vivo uptake and translocation of styrene maleic anhydride nano-particles in the stem and root area of *Murraya exotica* [34]. Likewise, Jiang et al. found that polystyrene nano-plastics with 100 nm sizes can be abundantly accumulated in the apical region of *Vicia faba* which blocked the connections of cells or cell wall generally used for nutrients transportation [35]. Besides, phytotoxic effects of MPs on other terrestrial plants such as lettuce (*Lactuca sativa*), perennial ryegrass (*Lolium perenne*), *Arabidopsis thaliana* were also reported in previous literature [36,37,38].

Land soil is an important source and main sink of MPs [39]. Most of the earlier studies on plant–soil interactions were based on the effect of plants on biological, chemical and physical properties of soil; effects of soil biological (microbial) properties on growth, diversity and community composition of plants [37,40]. In recent years, MP residues have shown a great threat to plant–microbe–soil systems, resulting in adverse effects on soil microorganisms, which includes reduction in rate of reproduction, increased neurotoxicity and cytotoxicity, induction of oxidative stress, and physical damage [41,42]; soil enzymes like urease, glucosidase, and phosphatase; change in leaf elemental (N and C content) composition [43]. On the other hand, Fei et al. [44] observed positive impact of polyvinyl chloride (PVC) and polyethylene (PE) MPs on acid phosphatase and urease activities in the soil. The negative effects of polypropylene and PE MPs on soil enzyme activities can increase FDAse activities in loess soil [45] while urease activities in cinnamon lake soils [46], respectively. MPs can alter soil enzyme activity via change in soil physical properties, direct adsorption and the surrounding microenvironment [47,48,49]. MP residues can impact bacterial activity and community composition by effecting nutrient contents of the soil; nutrients required for chlorophyll synthesis; dissolved organic matter [50,51] and relative abundance and community structure of arbuscular mycorrhizal fungi and nitrogen-fixing bacteria [52,53]. The mycorrhizal symbiosis is beneficial for plant growth and has contributed to the increase in plants biomass [54]. Additionally, MPs can alter the water cycling pattern, which directly influence the nutrients availability either by altering chemical speciation processes within soils or by distressing the activity of soil microbes.

Up to now, the researches on the physiological toxicity of MPs have been concentrated on aquatic organisms, although there is a huge lack of experiments probing into the MPs effects on the different growth indexes of terrestrial plants [38,39]. Seed germination is a key period of plant life cycle, which is more sensitive to external environmental factors and toxicity [37]. Therefore, seed germination can be used as an important indicator to evaluate the toxicological effects of MPs on terrestrial plants [37,55]. In the terrestrial ecosystem, plants are producers and main food sources for humans [56]. MPs can be quick to be absorbed, accumulated, biomagnified and transferred to a plant, which leads to grave toxicity to human health through food chain and many other exposure pathways [55,56]. Mung bean and soybean are the main food crops which are widely planted around the world [57,58]. Therefore, it will be of great environmental significance to explore the phyto-toxic effects of MPs on mung bean and soybean. The present study investigates the accumulation and absorption of PE MPs of two particle sizes (6.5 μm and 13 μm) in mung bean and soybean. Here, two questions are raised and expounded:

(1) How MPs with different sizes (6.5 μm and 13 μm) affect seed germination characteristics of (a) soybean (*Glycine max*) and (b) mung bean (*Vigna radiata*)?
(2) What are the differences between two studied species in response to applied MPs?

## 2. Materials and Methods

### 2.1. Preparation of Suspensions of MPs

For the present study, PE-MPs with particle size of 6.5 μm and 13 μm were selected. PE-MPs (250 mg/10 mL solution) were obtained from Tianjin BaseLineChromTech Research Centre located in Tianjin city, PR China. In this experiment, six concentration gradients were set for each particle size and each concentration had 6 repetitions. The concentration of MPs suspensions were designed as 0 mg/L (control), 10 mg/L, 50 mg/L, 100 mg/L, 200 mg/L, and 500 mg/L. To reduce the aggregation, MPs suspensions contained in beakers were sonicated for 1.5 h (25 °C, 40 kHz) prior to germination test. Glass bars were also used to stir the suspensions to avoid aggregation of the MPs particles. After sonication, MPs were homogeneously dispersed in the aqueous phase and the suspensions of different concentrations were stored in clean beakers.

### 2.2. Germination Test

The tested seeds of soybean and mung bean were purchased from a local market in Kaifeng City, Henan Province, China. Germination test was performed according to protocol of Xin et al. [59] with slight modifications. The seeds were soaked in 2% (*v/v*) sodium hypochlorite solution for 30 min to minimize the chances of microbial contamination and then rinsed with deionized water for three times to remove the residual solution. After this, seeds of equal size and with full grain were selected and placed in 9 cm Petri dishes (10 seeds/dish) with two pieces of filter papers at the bottom. To the Petri dishes, 10 mL of MPs suspension from each concentration was added and Petri dishes were held in a growth incubator (12 h/12 h day/night cycle) at 25 °C and 60% relative humidity. Every morning at 8:00 a.m., the number of germinated seeds were counted and recorded daily based on 2 mm radicle emergence (the criterion for germination was that the root length exceeded half of the seed length) after 3rd day and 7th day. After each record, 2 mL ultrapure water was added into the culture dish with a pipette gun in order to ensure sufficient water in the Petri dishes. On the 3rd and the 7th day, the root and shoot length of the seedlings in each sample was measured. After the measurement of root length, the fresh weight of seedlings was measured in time. Subsequently, seedlings were weighted after being dried in the oven at 105 °C for 24 h to constant mass. The seed vigor indices (germination rate, germination energy, germination index, vigor index, and mean germination speed), morphological index (root length and shoot length), and dry weight were calculated to explore the effect of PE-MPs on mung bean and soybean seed germination and seedling growth. The formulae used to calculate seed-vigor indices are shown in Table 1.

### 2.3. Statistical Analysis

For each concentration, the results were input and processed by Excel 2013 and presented as mean ± SD (standard deviation). IBM SPSS 22.0 (IBM, Armonk, NY, USA) was used to conduct one-way analysis of variance (ANOVA), and LSD or Dunnett’s T3 test was used for post multiple comparison. Origin 2019b software (Origin, Northampton, MA, USA) was used to draw bar charts.

## 3. Results and Discussion

### 3.1. Effect of PE-MPs on Seed Vigor of Soybean and Mung Bean

The effect of PE-MPs on soybean and mung bean seed vigor was described by germination rate, germination energy, germination index (germination index is the index of seed vigor, and higher the germination index, higher the vigor is), vigor index (vigor index is the comprehensive reflection of seed germination rate and growth) and mean germination speed.

As compared with 13 µm, 6.5 µm MPs size showed more obvious significant inhibition on germination energy and germination index of soybean seeds and the mean germination speed was found to be longer (Table 2). When the MPs concentration was medium, the effect on the growth characteristics of soybean seeds was more obvious, while the effect of MPs at low or high concentration was less.

For mung bean seeds (Table 3), the results showed no significant differences in germination energy, germination index, vigor index and germination rate among different concentrations of PE-MPs, but differences were observed in mean germination speed. The mean germination speed of mung bean seeds was significantly different (*p* < 0.05) under the treatment of 13 µm MPs, and the germination speed of the control group was slower than that of other groups. When mung bean seeds were exposed to different concentrations of 6.5 µm PE-MPs, no significant differences in germination energy, germination index, vigor index, mean germination speed and germination rate were observed. Vigor index showed no significant differences with respect to MP size and concentration.

#### 3.1.1. Effect of PE-MPs on Germination Energy of Soybean and Mung Bean

**Figure 1:**
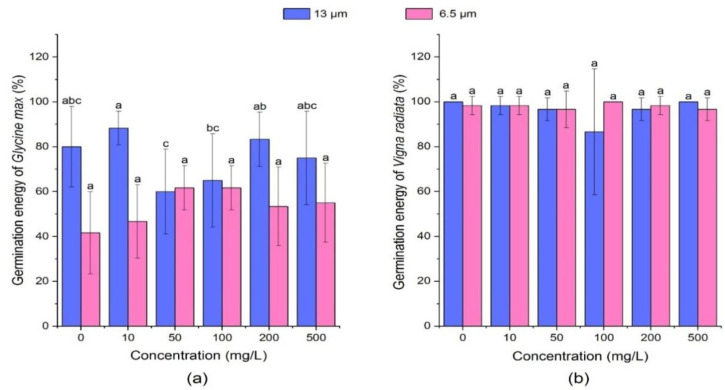
Effect of PE-MPs of different sizes and concentrations on seed germination energy. The letter a, b or c shows a significant difference between treatments with different MPs concentrations at the same particle type and size (*p* < 0.05).

The germination energy refers to the germination ratio within the specified date, i.e., 3 days in this experiment. The magnitude of germination energy indicates the germination percentage and the intensity of seed vigor (%). As shown in Figure 1, in soybean seeds when treated with 13 µm size MPs at a low concentration of 10 mg/L, a slight promoting effect on the germination energy of soybean sprouts existed, whilst other concentrations (0 mg/L, 50 mg/L, 100 mg/L, 200 mg/L, and 500 mg/L) showed inhibitory effect on germination energy. For instance, at concentrations of 50 mg/L and 100 mg/L, germination energy decreased by 20% and 15% as compared to the control, respectively. Different concentrations of 6.5 µm PE-MPs promoted the germination energy of soybean seeds. Compared with the blank control, the germination energy of soybean seeds increased in all treatment groups, especially at the concentrations of 50 mg/L and 100 mg/L. PE-MPs of both the particle sizes (6.5 and 13 µm) and concentrations (0 mg/L, 10 mg/L, 50 mg/L, 100 mg/L, 200 mg/L, and 500 mg/L) showed no significant effect (*p* > 0.05) on the germination energy of mung bean seeds. The promoting effect of MP might be due to structure of seed capsule of both soybean and mung bean seeds, which may protect it against action of polyethylene MP particles and controls germination while inhibitory effect of MP can be due to its accumulation in the seed capsule pores as observed in *Arabidopsis* seeds [60]. Bosker et al. [61] observed a delay in the germination rate of *L. sativum* due to blockage of the seed capsule with MP particles. MPs deposits on the surface of *Glycine max* pores showed slow water and nutrient uptake thereby delay in germination which might be due to physical blocking [61].

#### 3.1.2. Effect of PE-MPs on Germination Energy of Soybean and Mung Bean

**Figure 2:**
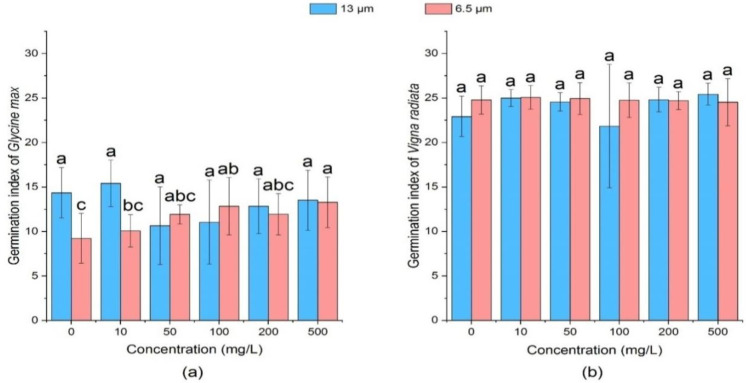
Effect of PE-MPs of different sizes and concentrations on seed germination index. The letter a, b or c shows a significant difference between treatments with different MPs concentrations at the same particle type and size (*p* < 0.05).

Germination index is the sum of the number of seeds germinated per day divided by the number of days. Figure 2 showed that 13 µm at 10 mg/L concentration had a slight promoting effect on germination index of soybean. However, germination index decreased at concentrations of 50 mg/L, 100 mg/L, 200 mg/L, and 500 mg/L. Particularly, the medium concentrations (50 mg/L and 100 mg/L) revealed a significant (*p* < 0.05) inhibitory effect (10.65 and 11.05) on the germination index, respectively. It was observed that all treatment groups of 6.5 µm PEs promoted the germination index of soybean seeds. At the concentration of 10 mg/L, the germination index had a small increase by 0.85. Compared with the control group, the germination index of soybean seeds was higher in all treatment groups. It was observed that medium concentrations (50 mg/L and 100mg/L) and high concentrations (200 mg/L and 500 mg/L) could significantly promote the germination index of soybean seeds.

For mung bean seeds, effect of MPs of all particle sizes and concentrations on the germination index was not significant (*p* > 0.05) when compared with the control group. It was found that mung bean’s germination indices of 13 µm size PE-MPs were smaller than those of 6.5 µm, except at high concentrations (200 mg/L and 500 mg/L). Further, 13 µm PE-MPs showed slight fluctuation in the germination index while that of 6.5 µm PE-MPs index was relatively stable. Seed germination is generally promoted by creation of pores for better uptake of water, improvement of antioxidative enzyme system, hydroxyl radicals generation for cell wall loosening and enhancement of starch hydrolysis [62,63]. Xin et al. [59] in their study observed improvement in the seed germination index due to higher water uptake induced by poly-succinimide nano-particles through new pores generation on the outer surface of seed coat. Additionally, water-soluble derivatives (amino acids) of micro/nano-particles hydrolysis can be useful in the protein biosynthesis and can be taken up by seeds as an energy and nutrient source [64].

#### 3.1.3. Effect of PE-MPs on Mean Germination Speed of Soybean and Mung Bean

**Figure 3:**
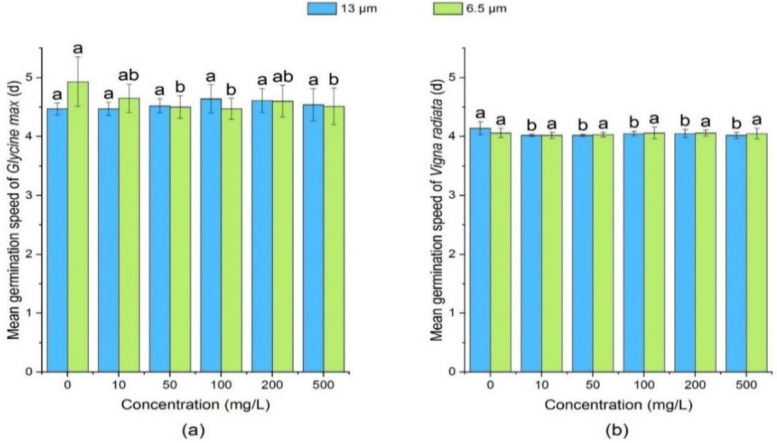
Effect of PE-MPs of different sizes and concentrations on mean germination speed. The letter a–c shows a significant difference between treatments with different MPs concentrations at the same particle type and size (*p* < 0.05).

Mean germination speed is an index which measures the rate of germination, and the smaller the value, the faster the germination speed is. Compared with the control group, 13 µm treatment groups showed no significant effect on the mean germination speed of soybean (Figure 3). The mean germination speed of soybean decreased under the treatment of 6.5 µm PE-MPs. As compared to the control, the mean germination speed of mung bean seeds was not significantly affected (*p* > 0.05) by different concentrations of 6.5 µm PE-MPs. This could be due to the nature of the MPs themselves, which can reduce the plant’s accessibility to MPs after aggregation [55].

#### 3.1.4. Effect of PE-MPs on Germination Rate of Soybean and Mung Bean

Germination rate (%) is an important indicator of the effect of MPs on seeds and it represents the percentage of the number of normal seedlings growing in a specified period to the number of tested seeds. High germination rate indicates that there are more live seeds, and vice versa.

**Figure 4:**
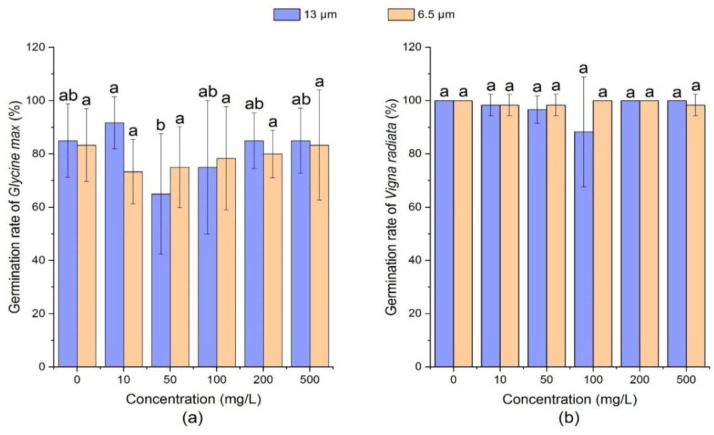
Effect of PE-MPs of different sizes and concentrations on germination rate. The letter a–c shows a significant difference between treatments with different MPs concentrations at the same particle type and size (*p* < 0.05).

Figure 4 showed that 13 µm PE-MPs at medium concentrations (50 mg/L and 100 mg/L) significantly declined the germination rate of soybean seeds (*p* < 0.05). For example, on the 7th day, the mean germination rate of soybean decreased by 20%. Under the treatment of different concentrations of 6.5 µm size PE-MPs, the germination rate of soybean declined to 75% (10 mg/L) and then increased 83.3% (500 mg/L), which was equivalent to the control (83.3%).

After exposure of mung bean seeds to PE-MPs, the germination speed was observed to range from 88.33 to 100 %. Regarding different particle sizes and different concentrations of PE-MPs, no significant effect on the germination rate of mung bean was observed as compared with the control. Bosker et al. confirmed that nano- and microplastics (50, 500, 4800 nm) can reduce the germination rate of cress (*Lepidium sativum*) and the adverse effect increased with plastic size [33]. Additionally, Pignattelli et al. conducted a study proving the inhibitory effect of PE-MPs on *Lepidium sativum*, and the percentage inhibition of germination of 55% [65].

### 3.2. Effect of PE-MPs on the Growth of Soybean and Mung Bean

All the concentrations of 13 µm PE-MPs inhibited the germination rate of soybean seeds and the strongest inhibitory effect was observed at the high concentration of 500 mg/L (Table 2). PE of 6.5 µm promoted the growth of soybean roots at each treatment group. When the concentration of both size PE-MPs was high, the adverse effect on soybean seedling was found to be stronger. PE-MPs of 13 µm size showed no apparent effect on the dry weight of soybean sprouts on the 7th day as compared with the control.

The root length (3rd and 7th day) and shoot length (3rd and 7th day) of mung bean in medium and high concentration (100, 200 and 500 mg/L) groups was generally longer than that of control group under the influence of different particle size PE-MPs (Table 3). Moreover, the exposure of 13 µm PE-MPs showed no adverse effect on the dry weight of mung bean seeds on the 3rd and 7th day, while only concentration of 100 mg/L PE-MPs reduced the dry weight which was 0.45 g (3rd day) and 0.32 g (7th day).

#### 3.2.1. Effect of PE-MPs on the Root Length and Shoot Length

**Figure 5:**
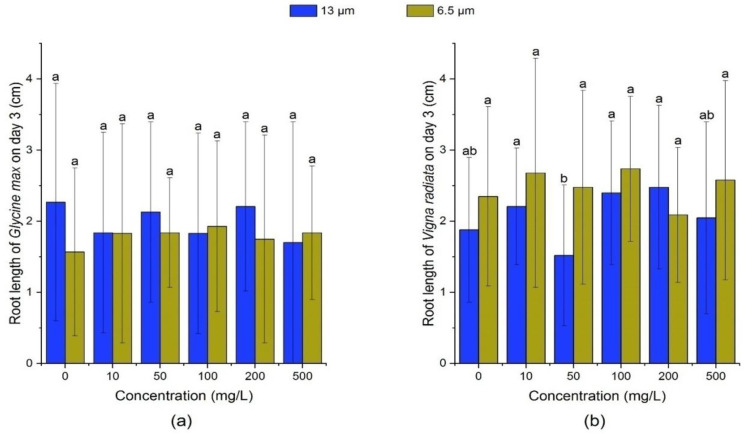
Effects of 13 and 6.5 μm PE-MPs on root length of *Glycine max* and *Vigna radiate* on the 3rd day. The letter a–c shows a significant difference between treatments with different MPs concentrations at the same particle type and size (*p* < 0.05).

On the 3rd day, there was no significant change (*p* > 0.05) in soybean root length under different concentrations and particle sizes of PE-MPs. Based on Table 2 and Figure 5, on the 7th day, the growth of soybean root was inhibited by 13 μm PE-MPs at different concentrations and the inhibition rate reached 30.77% at 500 mg/L. At 13 μm-100 mg/L and 6.5 μm-500 mg/L groups, PE-MPs could significantly promote the growth of soybean roots (*p* > 0.05).

**Figure 6:**
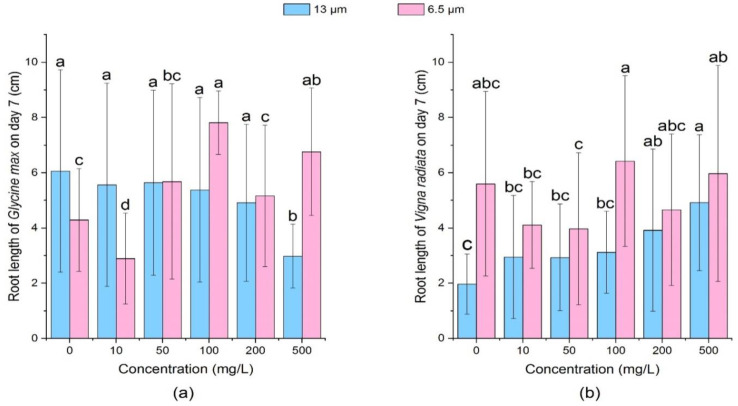
Effects of 13 and 6.5 μm PE-MPs on root length of *Glycine max* and *Vigna radiate* on the 7th day. The letter a, b or c shows a significant difference between treatments with different MPs concentrations at the same particle type and size (*p* < 0.05).

It was observed that 13 μm PE-MPs significantly promoted mung bean root length on the 7th day (*p* < 0.05), and the promotion effect was positively correlated with the concentration of MPs. On the 7th day, under the treatment of 6.5 μm PE-MPs, root length of mung beans was inhibited at the concentrations of 10, 50 and 200 mg/L, and promoted at the concentrations of 100 and 500 mg/L. As shown in Table 3 and Figure 6, root length of mung beans treated by 6.5 μm-100 mg/L PE-MPs suspension had the greatest promoting effect, which was 0.82 cm longer than that of effect on the 7th day.

**Figure 7:**
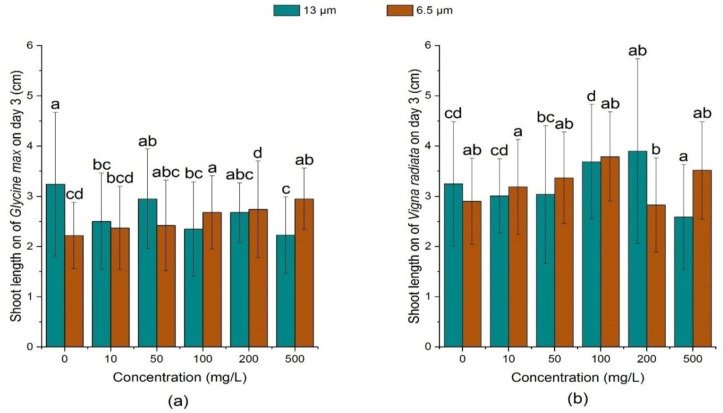
Effects of 13 μm and 6.5 μm PE-MPs on shoot length of *Glycine max* and *Vigna radiata* on the 3rd day. The letter a, b or c shows a significant difference between treatments with different MPs concentrations at the same particle type and size (*p* < 0.05).

**Figure 8:**
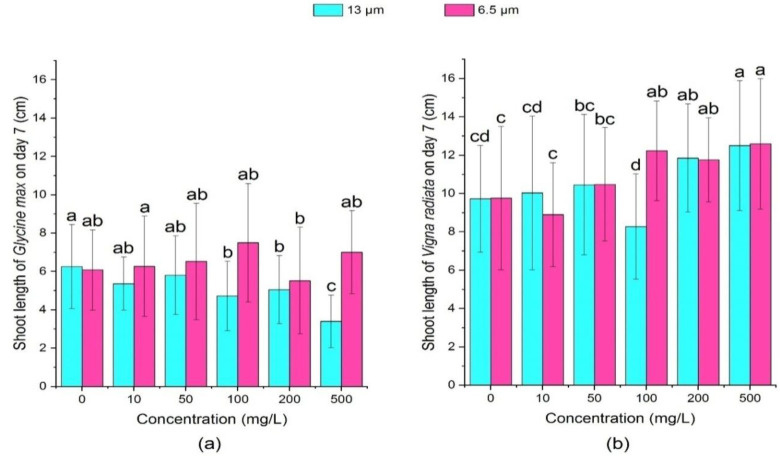
Effects of 13 μm and 6.5 μm PE-MPs on shoot length of *Glycine max* and *Vigna radiate* on the 7th day. The letter a, b or c shows a significant difference between treatments with different MPs concentrations at the same particle type and size (*p* < 0.05).

Figure 7 and Figure 8 shows that all concentrations of 13 μm PE-MPs could inhibit the growth of soybean seedlings on day 3 and day 7. Among them, the inhibition effect increased with the growth of MPs concentration, and soybean seedlings of high concentration (500 mg/L) treatment had the shortest seedling length. Compared with the large particle size (13 μm) treatment group, the small particle size (6.5 μm) could promote the growth of soybean sprouts, and there was a positive correlation between shoot length and PE-MPs concentration. On day 7, except 200 mg/L, all concentrations of 6.5 μm PE-MPs treatments promoted the growth of soybean shoot, whilst 13 μm PE-MPs hindered the growth of shoot of soybean at all concentrations.

Both the particle sizes of PE-MPs could promote the growth of mung bean sprouts at different concentrations, but the promotion effect was different on the 3rd day and the 7th day. On the 3rd day, 13 μm PE-MPs significantly increased (*p* < 0.05) the length of shoots of mung bean seedlings at the medium concentrations (100 mg/L and 200 mg/L). In contrast, on the 7th day, except 100 mg/L, 13 μm PE-MPs could significantly promote (*p* < 0.05) mung beans’ shoot length when the concentration was greater than 50 mg/L.

MPs can enhance the adsorption and reactivity of plants to pollutants, and change the pH, salinity, nutrient elements, organic matter and ion states of the environment [58]. Therefore, MPs change overall environment nutrient transfer, which results in negative effect on plant growth [66]. In addition, MPs provide adsorption sites for environmental microorganisms. A large number of different bacteria accumulate on the surface of MPs, which makes them more toxic, further reducing microbial abundance and microbial activity, destroying microbial diversity and microbial environment, and affecting the ecological environment required for plant growth [67]. The present study showed similar results with the study of Bosker et al. [33], which attributed the root and shoot growth inhibitory effect of MPs to inhibition of water uptake via transient short term mechanical blockage of seed pores, while with passage of time root and shoot length promoting effect was also observed. Additionally, increase in seed germination, seedling growth and root root elongation of *Triticum aestivum,* but consquently, reduction in shoot to root biomass ratio was was observed after 5 days treatment of polystyrene nanoplastics [68].

#### 3.2.2. Effects of PE-MPs on the Dry Weight

**Figure 9:**
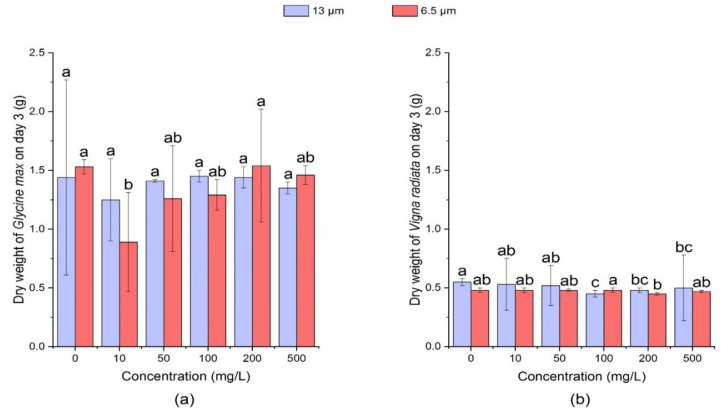
Effects of 13 μm and 6.5 μm PE-MPs on dry weight of *Glycine max* and *Vigna radiata* on the 3rd day. The letter a, b or c shows a significant difference between treatments with different MPs concentrations at the same particle type and size (*p* < 0.05).

**Figure 10:**
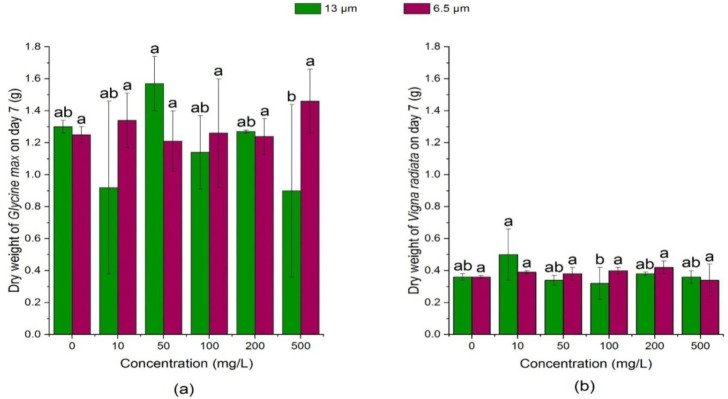
Effects of 13 μm and 6.5 μm PE-MPs on dry weight of *Glycine max* and *Vigna radiata* on the 7th day. The letter a–c shows a significant difference between treatments with different MPs concentrations at the same particle type and size (*p* < 0.05).

Figure 9 and Figure 10 showed that on the 3rd day, the dry weight of soybean sprouts was not significantly affected (*p* > 0.05) by the high concentration of 13 μm PE-MPs (200 mg/L and 500 mg/L), but it was significantly reduced (*p* < 0.05) at low concentrations (10 mg/L, 50 mg/L) and medium concentration (100 mg/L) as compared with the control. On the 7th day, 13 μm PE-MPs had no significant effect (*p* > 0.05) on the dry weight of soybean seedlings. In addition, on the 3rd day, the dry weight of soybean in the 6.5 μm PE-MPs treatment group was similar to that in the 13 μm treatment group on the 3rd day.

It was found that the exposure of 6.5 μm PE-MPs had no significant effect (*p* > 0.05) on the dry weight on the 3rd and 7th day. Further, as compared with the control, the dry weight of 13 μm PE-MPs at the concentration of 100 mg/L on the 3rd and 7th day was significantly reduced (*p* < 0.05). When MPs reach sub-micron and micron level in the environment, it enters into the stems and leaves of the plant through the root system and then accumulate inside resulting in damage to the photosynthetic process, blocking the stoma of the plant cell wall, inhibiting water absorption and affecting the transportation of nutrients thereby directly affect the growth of plant [65]. Human consumption of plants will bring potential risks to the human body affecting human health and development. Some current studies have shown that MPs have their respective effects on physical and chemical properties of the environment, plant growth, animal growth and development and microbial and enzyme activities, but the relevant mechanisms underlying toxicity mechanism are still unclear [69]. Machado et al. [32] reported a similar trend of dry weight in *Allium fistulosum* where increase in root dry weight under the treatment of polystyrene type MP while a decrease in weight under polypropylene was observed. Additionally, polyamide MP treatment showed a decrease in root: shoot dry weight, while polypropylene MP showed an increase in the ratio.

## 4. Conclusions

The present research highlighted that different sizes of polyethylene MPs can affect the germination and growth of soybean (*Glycine max*) and mung bean (*Vigna radiata*) during chronic exposure. Importantly, the detrimental impacts brought by polyethylene MPs to soybean were more serious than that to mung bean at the studied concentrations. In contrast to the unapparent effect of PE-MPs on mung bean, germination energy, germination index, and vigor index of soybean were decreased when exposed to PE-MPs. In addition, 13 µm PE-MPs supplied in this study were capable to restrain the growth of the root length and shoot length of soybean seedlings, and inhibitory effects of PE-MPs happened when crops were exposed to 6.5 µm PE-MPs. Based on the present results, 13 µm PE-MPs at low and medium concentrations (10, 50, and 100 mg/L) showed strong inhibition of soybean growth. Overall, our results suggested the significance of exploring the harmful impact of PE-MPs on soybean and mung bean, which are the main food sources for human beings.

## Figures and Tables

**Table 1:** Formulae for Seed vigor (%) indices calculation.

Index	Formula
Germination Rate (GR)	$\mathrm{GR}\;=\left(\frac{N_{7d}}{N_{t}}\right)\times 100\%$
Germination Index (GI)	$\mathrm{GI}=\sum \frac{G_{i}}{D_{i}}$
Mean Germination Time (MGT) (d)	$\mathrm{MGT}=\frac{\sum \left(D_{i}\times G_{i}\right)}{\sum G_{i}}$
Germination Energy (GE)	$\mathrm{GE}=\frac{N_{3d}}{N_{t}}\times 100\%$

*N_t_* was the total number of tested seeds and *N*_3d_ & *N*_7d_ were the number of seeds on day 3 and day 7, respectively. *D_i_* represented the ith day of germination, S is the seedling height of the 7th day, *G_i_* is the number of germinated seeds corresponding to *D_i_* and d represents number of days.

**Table 2:** Effect of PE-MPs on the growth of soya bean (*Glycine max*) seedlings.

PE (µm)	Concentration (mg/L)	Root Length on Day 3 (cm)	Root Length on Day 7 (cm)	Shoot Length on Day 3 (cm)	Shoot Length on Day 7 (cm)	Dry Weight on Day 3 (g)	Dry Weight on Day 7 (g)
13	0	2.27 ± 1.67 ^a^	6.05 ± 5.21 ^a^	3.24 ± 1.43 ^a^	6.25 ± 2.19 ^a^	1.44 ± 0.83 ^a^	1.30 ± 0.04 ^a^
	10	1.84 ± 1.41 ^a^	5.56 ± 5.41 ^ab^	2.50 ± 0.96 ^bc^	5.36 ± 1.39 ^ab^	1.25 ± 0.35 ^a^	0.92 ± 0.54 ^a^
	50	2.13 ± 1.27 ^a^	5.64 ± 5.42 ^ab^	2.95 ± 0.99 ^ab^	5.80 ± 2.05 ^ab^	1.41 ± 0.01 ^a^	1.57 ± 0.77 ^a^
	100	1.83 ± 1.41 ^a^	5.38 ± 5.16 ^ab^	2.35 ± 0.94 ^bc^	4.72 ± 1.81 ^b^	1.45 ± 0.05 ^a^	1.14 ± 0.23 ^a^
	200	2.21 ± 1.19 ^a^	4.91 ± 3.46 ^ab^	2.68 ± 0.59 ^abc^	5.05 ± 1.78 ^b^	1.44 ± 0.09 ^a^	1.27 ± 0.01 ^a^
	500	1.70 ± 1.35 ^a^	3.02 ± 1.45 ^b^	2.23 ± 0.76 ^c^	3.40 ± 1.37 ^c^	1.35 ± 0.05 ^a^	0.90 ± 0.54 ^a^
6.5	0	1.57 ± 1.18 ^a^	4.29 ± 2.91 ^bc^	2.22 ± 0.66 ^b^	6.08 ± 2.09 ^ab^	1.53 ± 0.06 ^a^	0.92 ± 0.57 ^a^
	10	1.83 ± 1.54 ^a^	2.99 ± 3.02 ^c^	2.37 ± 0.83 ^ab^	6.26 ± 2.62 ^a^	0.89 ± 0.42 ^b^	1.34 ± 0.17 ^a^
	50	1.84 ± 0.77 ^a^	5.68 ± 4.98 ^abc^	2.42 ± 0.90 ^ab^	6.52 ± 3.03 ^ab^	1.26 ± 0.45 ^ab^	1.34 ± 0.29 ^a^
	100	1.38 ± 1.46 ^a^	7.84 ± 5.34 ^a^	2.68 ± 0.73 ^ab^	7.50 ± 3.09 ^ab^	1.29 ± 0.13 ^ab^	1.29 ± 0.39 ^a^
	200	1.70 ± 1.46 ^a^	5.16 ± 4.60 ^abc^	2.74 ± 0.96 ^ab^	5.52 ± 2.78 ^b^	1.54 ± 0.48 ^a^	1.24 ± 0.11 ^a^
	500	1.84 ± 0.94 ^a^	6.80 ± 3.96 ^ab^	2.95 ± 0.61 ^a^	7.00 ± 2.17 ^ab^	1.46 ± 0.08 ^ab^	1.40 ± 0.09 ^a^

The letter a–c shows a significant difference between treatments with different MPs concentrations at the same particle type and size (*p* < 0.05).

**Table 3:** Effect of PE-MPs on the growth of mung bean (*Vigna radiata*) seedlings.

PE (µm)	Concentration (mg/L)	Root Length on day 3 (cm)	Root Length on Day 7 (cm)	Shoot Length on Day 3 (cm)	Shoot Length on Day 7 (cm)	Dry Weight on Day 3 (g)	Dry Weight on Day 7 (g)
13	0	1.88 ± 1.02 ^ab^	1.97 ± 1.09 ^c^	3.25 ± 1.24 ^bc^	9.73 ± 2.79 ^cd^	0.55 ± 0.03 ^a^	0.36 ± 0.02 ^ab^
	10	2.21 ± 0.82 ^a^	2.95 ± 2.23 ^bc^	3.01 ± 0.74 ^cd^	10.03 ± 4.01 ^cd^	0.53 ± 0.22 ^ab^	0.50 ± 0.16 ^a^
	50	1.52 ± 0.99 ^b^	2.93 ± 1.93 ^bc^	3.04 ± 1.37 ^cd^	10.46 ± 3.66 ^bc^	0.52 ± 0.17 ^ab^	0.34 ± 0.33 ^ab^
	100	2.4 ± 1.01 ^a^	3.12 ± 1.48 ^bc^	3.69 ± 1.14 ^ab^	8.28 ± 2.75 ^d^	0.45 ± 0.03 ^c^	0.32 ± 0.10 ^b^
	200	2.48 ± 1.15 ^a^	3.92 ± 2.93 ^ab^	3.90 ± 0.84 ^a^	11.85 ± 2.82 ^ab^	0.48 ± 0.02 ^bc^	0.38 ± 0.01 ^ab^
	500	2.05 ± 1.35 ^ab^	4.92 ± 2.46 ^a^	2.59 ± 1.04 ^d^	12.50 ± 3.38 ^a^	0.50 ± 0.28 ^bc^	0.36 ± 0.04 ^ab^
6.5	0	2.35 ± 1.26 ^a^	5.60 ± 3.34 ^abc^	2.90 ± 0.86 ^cd^	9.76 ± 3.74 ^c^	0.48 ± 0.02 ^ab^	0.36 ± 0.01 ^a^
	10	2.68 ± 1.61 ^a^	4.11 ± 1.57 ^bc^	3.19 ± 0.94 ^bcd^	8.90 ± 2.71 ^c^	0.48 ± 0.02 ^ab^	0.39 ± 0.01 ^a^
	50	2.48 ± 1.36 ^a^	3.97 ± 2.76 ^c^	3.37 ± 0.91 ^abc^	10.48 ± 2.95 ^bc^	0.48 ± 0.01 ^ab^	0.38 ± 0.04 ^a^
	100	2.74 ± 1.02 ^a^	6.42 ± 3.09 ^a^	3.79 ± 0.89 ^a^	12.23 ± 2.60 ^ab^	0.48 ± 0.02 ^a^	0.40 ± 0.02 ^a^
	200	2.09 ± 0.95 ^a^	4.66 ± 2.74 ^abc^	2.83 ± 0.94 ^d^	11.76 ± 2.20 ^ab^	0.45 ± 0.01 ^b^	0.42 ± 0.04 ^a^
	500	2.58 ± 1.40 ^a^	5.97 ± 3.91 ^ab^	3.52 ± 0.97 ^ab^	12.59 ± 3.40 ^a^	0.47 ± 0.01 ^ab^	0.34 ± 0.10 ^a^

The letter a–c shows a significant difference between treatments with different MPs concentrations at the same particle type and size (*p* < 0.05).

## Data Availability

Data is contained within the article.
